# SIGMAP: an explainable artificial intelligence tool for SIGMA-1 receptor affinity prediction†

**DOI:** 10.1039/d4md00722k

**Published:** 2024-11-08

**Authors:** Maria Cristina Lomuscio, Nicola Corriero, Vittoria Nanna, Antonio Piccinno, Michele Saviano, Rosa Lanzilotti, Carmen Abate, Domenico Alberga, Giuseppe Felice Mangiatordi

**Affiliations:** a Dipartimento di Medicina di Precisione e Rigenerativa e Area Jonica (DiMePRe-J), Università degli Studi di Bari Aldo Moro Piazza Giulio Cesare, 11, Policlinico 70124 Bari Italy; b CNR – Institute of Crystallography Via Amendola 122/o 70126 Bari Italy domenico.alberga@cnr.it giuseppefelice.mangiatordi@cnr.it; c Department of Computer Science, University of Bari “Aldo Moro” Via E. Orabona, 4 I-70125 Bari Italy; d CNR – Institute of Crystallography Via Vivaldi 43 81100 Caserta Italy; e Department of Pharmacy – Pharmaceutical Sciences, University of Bari “Aldo Moro” Via E. Orabona, 4 I-70125 Bari Italy

## Abstract

Developing sigma-1 receptor (S1R) modulators is considered a valuable therapeutic strategy to counteract neurodegeneration, cancer progression, and viral infections, including COVID-19. In this context, *in silico* tools capable of accurately predicting S1R affinity are highly desirable. Herein, we present a panel of 25 classifiers trained on a curated dataset of high-quality bioactivity data of small molecules, experimentally tested as potential S1R modulators. All data were extracted from ChEMBL v33, and the models were built using five different fingerprints and machine-learning algorithms. Remarkably, most of the developed classifiers demonstrated good predictive performance. The best-performing model, which achieved an AUC of 0.90, was developed using the support vector machine algorithm with Morgan fingerprints. To provide additional, user-friendly information for medicinal chemists in the rational design of S1R modulators, two independent explainable artificial intelligence (XAI) approaches were employed, namely Shapley Additive exPlanations (SHAP) and Contrastive Explanation. The top-performing model is accessible through a user-friendly web platform, SIGMAP (https://www.ba.ic.cnr.it/softwareic/sigmap/), specifically developed for this purpose. With its intuitive interface, robust predictive power, and implemented XAI approaches, SIGMAP serves as a valuable tool for the rational design of new and more effective S1R modulators.

## Abstract 2

Developing sigma-1 receptor (S1R) modulators is considered a valuable therapeutic strategy to counteract neurodegeneration, cancer progression, and viral infections, including COVID-19.

## Graphical abstract

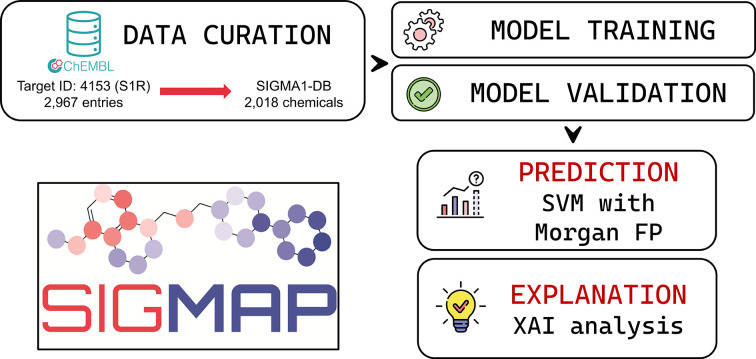

## 1. Introduction

Sigma receptors have intrigued scientists since their discovery in the 1970s.^1^ Initially, they were misclassified as subtypes of opioid receptors, but subsequently they were identified as a new class of proteins functionally and pharmacologically classified in two different subtypes, namely the sigma-1 receptor (S1R) and sigma-2 receptor (S2R).^2^ The two subtypes are localized in the central nervous system (CNS) and peripheral tissues and organs including the liver, kidneys and endocrine glands.^3,4^ In particular, S1R is predominantly expressed in endoplasmic reticulum–mitochondria associated membranes (MAMs) where it functions as a ligand-regulated chaperone protein modulating Ca^2+^ fluxes.^5^ Upon ligand binding, S1R translocates to modulate a number of proteins including ion channels and G-protein-coupled receptors.^6^ With its neuroprotective function, S1R is a target for the development of therapeutics against neurodegenerative diseases with drugs in advanced clinical phases for the treatment of Alzheimer's and Huntington's diseases as well as amyotrophic lateral sclerosis (ALS).^7^ In 2020, a study by Gordon *et al.*^8^ mapped the interactions between SARS-CoV-2 and human proteins, revealing that sigma receptor modulators may play a crucial role in treating COVID-19 infection. Specifically, the coronavirus proteins NSP6 and Orf9c were found to interact with S1R and S2R, respectively, suggesting these receptors as promising pharmacological targets in this context as well. In addition to the above reported neuroprotective actions, ligands binding to S1R are active against neuropathic pain and result in anti-proliferative and cytotoxic effects in a number of neoplastic cells.^9^ Because of its widespread importance, S1R is known as a ‘pluripotent chaperone’ and the discovery of new ligands capable of binding to S1R with high affinity has been actively pursued in the last few years. Traditional methods for assessing ligand affinity towards the receptor are frequently expensive and time-consuming, thereby hindering progress in research and the development of novel treatments. Commonly, radioactive receptor–ligand binding assays utilizing radioactively labeled ligands like [^3^H]-(+)-pentazocine are employed. These assays are based on the principle of competitive interaction between the labeled ligand and the analyte for the same receptor binding site.^10^ The strength of this technique is its exceptional sensitivity in detecting receptor–ligand binding, leveraging the radioactive properties of tritium to detect even minute quantities of binding. Other advantages are the specificity of (+)-pentazocine binding to S1R and the straightforward nature of the assay.^10^ However, the medium throughput^10^ of these assays, along with the large quantities of radiochemicals required, makes them relatively slow to perform and raises safety and disposal concerns.^11^ For this reason, the scientific community is focusing efforts towards the development of alternative experimental techniques. Among these, the use of fluorescent ligands as an alternative to radioligands looks very promising. Although a few fluorescent ligands specific for sigma receptors have been developed, the selectivity for the sigma-1 subtype needs to be improved for effective use in fluorescence-based techniques when assessing novel compounds.^12–14^ Moreover, although binding assays involving radioactive and fluorescent ligands are invaluable in studying ligand–receptor interactions when handling a small number of samples, their limitations hinder the discovery and development of new ligands based on the screening of a large number of compounds. For this reason, innovative approaches to be adopted even before chemical synthesis could be advantageous. Despite the growing importance of S1R in recent years and the abundance of available bioactivity data, machine learning has surprisingly never been used as a tool for screening and optimizing potential S1R ligands. Instead, other types of predictive models can be found in the literature. In 1994, Glennon *et al.* identified the first 2D pharmacophore model,^15^ making significant progress in rationalizing the development of S1R ligands and leading the scientific community to the generation of increasingly sophisticated 2D and 3D ligand-based pharmacophore models.^16–20^ These models consistently emphasize the presence of positive ionizable (PI) groups and multiple hydrophobic elements. Furthermore, except for the models developed by Glennon and Langer, they typically include a polar group.^21^ Exploiting the refinement in homology modeling techniques, the first S1R homology model was published in 2011,^22^ enabling the identification of a likely binding site. This advancement facilitated the implementation of docking-based virtual screening and binding-affinity determination studies for new potential S1R ligands. In 2016, the release of the first crystal structure of human S1R,^6^ also complexed with multiple compounds,^23^ provided invaluable insights. This structural information has been leveraged for docking studies and development of various 3D structure-based pharmacophore models,^13,21,24–26^ enhancing binding-affinity prediction in virtual screenings. However, an explainable intelligent system capable of supporting chemists in designing new potential ligands for S1R has never been developed. This work aims to fill this gap by providing an innovative tool to S1R research. Particularly, in the present study, 25 machine learning-based models predicting S1R ligand affinity were developed employing five classification algorithms: random forest (RF), *K*-nearest neighbors (*K*-NN), gradient boosting (GB), extreme gradient boosting (XGB) and support vector machine (SVM). These models were built using five different fingerprints: AtomPair,^27^ Morgan,^28^ MACCS,^29^ Torsion^30^ and CSFP^31^ to characterize the dataset (*SIGMA1-DB*), extracted from ChEMBL version (v) 33 and comprising 2018 compounds split into a training set (TS) and a validation set (VS). Our goal extends beyond merely predicting S1R affinity with the most effective model; we also aim to clarify the rationale behind these predictions. To this end, we integrated two independent explainable artificial intelligence (XAI) approaches: Shapley Additive exPlanations (SHAP)^32^ and Contrastive Explanation.^33^ Leveraging a methodology already successfully applied by our group,^34,35^ we have incorporated the best-performing model and the two XAI analyses into a user-friendly web platform named SIGMAP. This platform stands out by not requiring any expertise in cheminformatics, making it an invaluable resource for medicinal chemists seeking early evaluations of S1R affinity potential. To the best of our knowledge, SIGMAP is the first freely accessible tool that can efficiently predict the S1R affinity potential of drug candidates, combining advanced predictive capabilities with transparent and interpretable outputs.

## 2. Materials and methods

### 2.1 Dataset preparation

A total of 2967 entries, all annotated exclusively with *K*_i_ values, were extracted from the ChEMBL v33 database.^36^ This extraction was conducted based on the target ID (CHEMBL4153) assigned to the S1R protein having guinea pig (referred to as *Cavia porcellus* in the ChEMBL database) as the target organism. This strategy was followed to obtain a dataset that is as extensive and homogeneous as possible. It is worth noting that guinea pig S1R is considered a valuable model for studying human S1R in preclinical research.^14,37–41^ As a matter of fact, this protein is highly conserved among mammals, and specifically, a 93% amino acid sequence identity was observed between the human and guinea pig S1Rs.^42^ To ensure the quality of the data, we implemented a methodology similar to that successfully applied for other classifiers.^34,35,43^ The database underwent a meticulous filtering process to retain only entries that met the following specific criteria: i) being marked as tested with a “binding” assay type (‘assay_type’ = ‘B’), which indicates direct binding of the compound to the molecular target; ii) lacking any warnings in the ‘*data_validity_comment*’ field, and iii) not indicating ‘*Not Determined*’ in the ‘comment’ field. By applying these filters, the dataset was refined to 2896 compounds. Furthermore, we assessed the validity of each SMILES string using an in-house semiautomated procedure implemented in the KNIME platform.^44^ This procedure specifically enables the exclusion of organometallic and inorganic compounds and chemicals featuring uncommon elements and mixtures, the neutralization of salts, and the removal of stereochemistry information. Subsequently, the OpenBabel^45^ node integrated into KNIME facilitated the conversion of the retrieved SMILES into a standardized QSAR-ready format. For the sake of standardization, we converted *K*_i_ values from molar concentration (*M*) to p*K*_i_ (−log *K*_i_). Subsequently, duplicate entries were aggregated into unique records, and the mean and the standard deviation (*σ*) of the p*K*_i_ values were computed. Compounds with *σ* exceeding 2 (*i.e.*, 15 instances identified as outliers) were excluded from the study. In conclusion, by removing 863 duplicate entries, the final curated dataset comprises 2018 chemicals in SMILES format (referred to as *SIGMA1-DB*) along with their corresponding experimental p*K*_i_ mean values. Aiming at categorizing *SIGMA1-DB* into chemicals with high S1R affinity (S(+)) and low or absent S1R affinity (S(−)), we set a threshold of p*K*_i_ = 7. This resulted in 1102 positive and 916 negative samples.

### 2.2 Dataset splitting

**Fig. 1:**
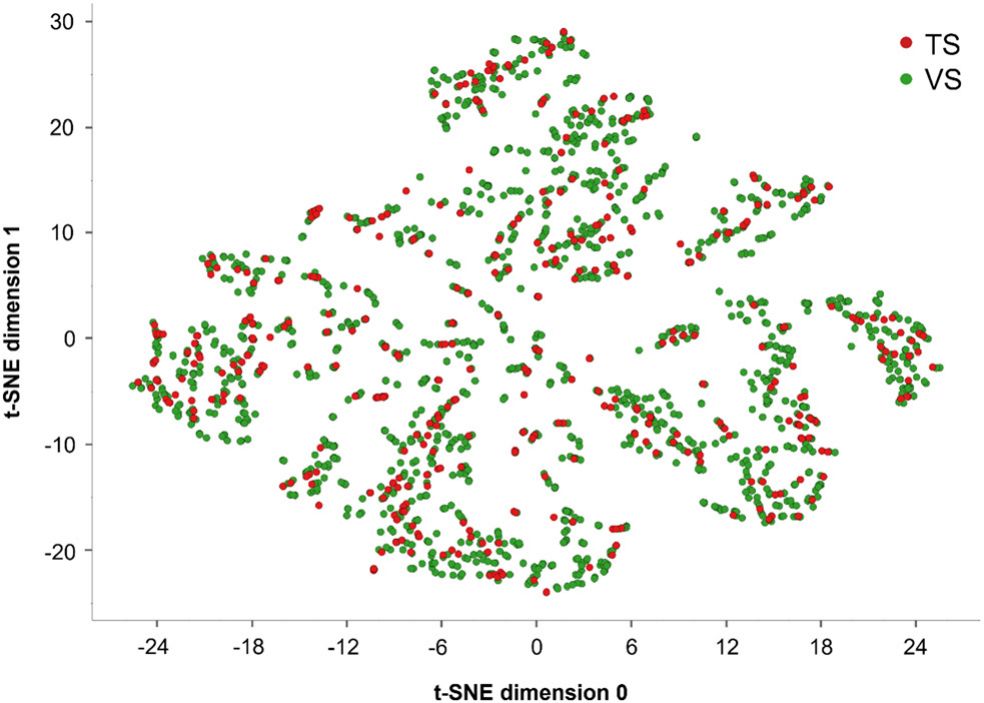
T-distributed stochastic neighbor embedding (t-SNE) analysis based on 9 physicochemical properties of the compounds belonging to the training set (TS), in red, and the validation set (VS), in green.

With the aim of splitting *SIGMA1-DB* into a TS and a VS, a rational approach was employed. Using the RDkit Diversity Picker node,^46^ the dataset was split into two classes, S(+) and S(−). This node automatically generates Morgan fingerprints^28^ for each SMILES string and then picks 80% of the most diverse molecules for each class based on the Tanimoto distance.^46^ This fingerprint is known to be one of the best performing in virtual screening procedures.^47^ In this way, a TS of 1615 compounds (80% of each class) and a VS including the remaining 403 compounds were built. Notice that an equal proportion of S(+) and S(−) compounds was maintained during the dataset splitting, with 882 S(+) and 733 S(−) in the TS and 220 S(+) and 183 S(−) in the VS*.* To illustrate the structural variability of the TS and the VS across the *SIGMA1-DB* dataset, a t-distributed stochastic neighbor embedding (t-SNE) analysis^48^ was performed based on 9 physicochemical properties of the molecules. These properties were calculated using the Canvas molecular descriptor KNIME node and then standardized using the Normalizer KNIME node (Fig. 1). The score plot of the first two t-SNE dimensions shows each ligand belonging to the two different datasets in the resulting 2D chemical space.

### 2.3 Development and validation

In this study, we employed five classification algorithms namely RF, *K*-NN, GB, XGB and SVM using the following KNIME nodes: tree ensemble learner, tree ensemble predictor, *K*-nearest neighbor, gradient boosted tree learner, gradient boosted tree predictor, XGBoost tree ensemble learner, XGBoost predictor, LibSVM (3.7) and Weka predictor (3.7).^49–52^ To represent each SMILES string in the dataset, we tested five different types of fingerprints aiming at identifying the best one for describing the chemicals in the *SIGMA-DB*. Specifically, using the RDKit Fingerprint KNIME node, we computed the following fingerprints for each chemical: i) ‘Atom pairs’ (1024 bits), based on the atomic environments and shortest path separations of every atom pair in the molecule;^27^ ii) ‘Morgan’ (radius 2–1024 bits), a circular Extended-Connectivity Fingerprint (ECFP4) based on the Morgan algorithm;^28^ iii) ‘MACCS’ (166 bits), a substructure key-based fingerprint^29^ and iv) ‘Torsion’ (1024 bits), based on the topological torsion descriptor.^30^ Moreover, we computed CSFP fingerprints^31^ using a python script reported by Bajorath *et al.*^53^ Following this approach, each chemical substructure was codified by five different binary representations to indicate the presence (1) or absence (0) of specific characteristics. As a first step, we identified the optimal setting (shown in Table S1 in the ESI†) through hyperparameter tuning performed on a 5-fold cross-validation (5-CV). Note that, for each algorithm, we considered the hyperparameters known to be responsible for the higher impact on the overall performance.^54,55^ To do that, we employed a grid search for *K*-NN where only two parameters were optimized and a Bayesian optimization for RF, GB, XGB and SVM to reduce the computational cost. Finally, after performance evaluation, we selected the best-performing model.

### 2.4 Applicability domain

When query chemicals differ significantly from the compounds used to train a QSAR (quantitative structure–activity relationship) model, the predictions it provides cannot be considered reliable. To address this issue and enhance confidence in the predictions, an applicability domain (AD) was established for the TS. The AD delineates the chemical space from which the models are built, thereby indicating where predictions are considered reliable.^56^ The similarity between a predicted chemical and the compounds in the TS is the main criterion for defining the structural domain of a QSAR model. Specifically, using the domain-similarity KNIME node, we calculated the Euclidean distances between the TS compounds and those being predicted. This approach defines an AD threshold (ADT) through the following steps: (i) calculating all Euclidean distances between all possible pairs of TS compounds, based on representative descriptors (in our case, Morgan fingerprints); (ii) creating a set of distances that are below the average distance calculated in step (i); (iii) computing the mean (*d*) and standard deviation (*σ*) of the distances in the set from step (ii); and (iv) defining the ADT (AD threshold) using the equation:

1 ADT = *d* + *Z*

where *Z* is an empirical cutoff value, set to 0.5 by default.^57^ The AD threshold determined for the TS in this study was 7.52.

### 2.5 Performance evaluation

We evaluated each classifier using Coopers statistics. Specifically, sensitivity (SE), specificity (SP), and accuracy (ACC) were computed as follows:2
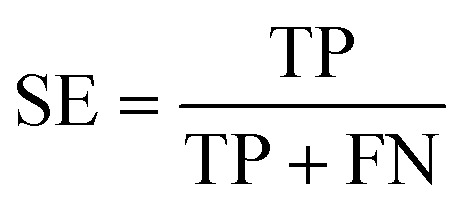
3
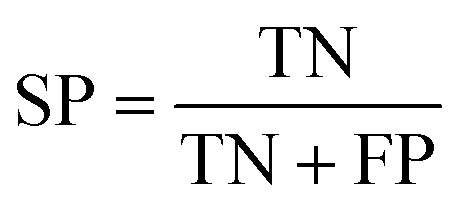
4
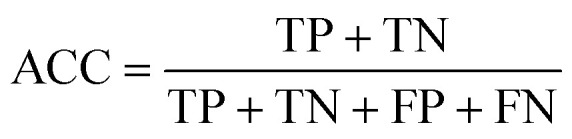
where TP (true positives) and TN (true negatives) represent correctly classified positive and negative samples, respectively, whereas FP (false positives) and FN (false negatives) are the misclassified positive and negative samples. Additionally, we evaluated the model performance through another quality metric, namely the Matthews correlation coefficient (MCC). This metric produces a high score only if the predictions return good results across all four confusion matrix categories (TP, FN, FN, and FP), considering both the size of positive elements and the size of negative elements in the dataset.^58^ The MCC ranges between −1 and +1, where +1 indicates perfect classification, 0 indicates a random classification, and −1 is a complete misclassification.

The formula for the MCC is as follows:5
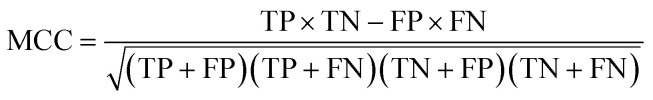
The area under the curve (AUC) was determined using the ROC curve node^59^ to assess the capability of the model to distinguish between S(+) and S(−) samples. This metric, which ranges from 0 (miss-classifiers) to 1 (ideal-classifiers), reflects the probability that positive compounds are ranked higher than decoys based on the prediction confidence values generated by the KNIME predictor nodes^49–52^ for each specific algorithm used. Finally, we computed and considered the positive (+LR) and the negative likelihood ratio (−LR). The classification model becomes more informative as the +LR value increases (or the −LR value decreases). Particularly, we will focus our attention on the +LR value which estimates how much the probability of a compound being S(+) increases relative to its initial probability, before any classification is performed.

The formulas are:6
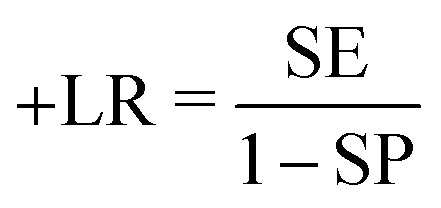
7
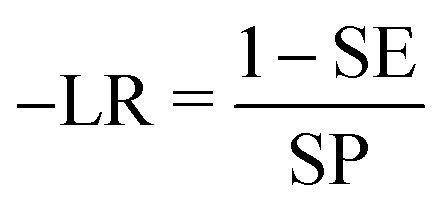


### 2.6 Explainability

Explainable artificial intelligence (XAI) is a branch of AI focused on developing tools to interpret “black-box” models. An explanation provides additional context and reasons behind one or more predictions,^60^ making models clearer, more transparent, and trustworthy. In this context, we implemented two different local *post hoc* XAI methods for distinct applications. Firstly, Shapley Additive exPlanations (SHAP) was used to explain which portions of the molecule most influence the prediction. SHAP is a feature attribution-based method that explains black-box predictions by assigning each input feature a numerical value, called the Shapley value, indicating its contribution to the prediction.^32^ Since in this work each molecule in the *SIGMA1-DB* is described in each model by one of the selected fingerprints (*i.e.*, AtomPair, Morgan, Torsion, MACCS, CSFP), the features correspond to the bits of the fingerprint, where each bit indicates the presence (1) or absence (0) of specific characteristics. SHAP quantifies the contribution of each molecular feature, where more positive (negative) contributions indicate a stronger influence of the corresponding bit towards a positive (negative) affinity prediction. This method offers a *complete*^32^ explanation by distributing the prediction value fractionally across all features. To calculate Shapley values, we used Shap loop start and Shap loop end KNIME nodes. However, Shapley values are descriptive but not *actionable* as they do not suggest how to change features to modify the output.^61^ To address this, we also implemented Contrastive Explanations, which provide users with both similar and dissimilar (counterfactual) examples.^61^ Similar examples can enhance the robustness of the classifier by confirming the ability of the model to remain stable within the chemical prediction space. Counterfactual examples, on the other hand, make this analysis *actionable* by suggesting small structural changes necessary to alter the prediction.^61,62^ This analysis was achieved by generating 10 structural analogs of the input using *DeLA-DrugSelf*,^63,64^ an in-house generative algorithm based on recurrent neural networks (RNN). The algorithm takes a SELFIES^65^ string as an input and generates a new molecule applying *N* mutations to the string (token substitutions, insertions or deletions) driven by the trained RNN. We set *N* = 1 and required a Tanimoto similarity of at least 0.5 for the generated compounds with respect to the query. Notice that the token of the SELFIES string chosen for the mutation is randomly selected.

### 2.7 Molecular docking simulations

Two selected compounds were docked on the X-ray structure of S1R in complex with PD144418 (PDB code: 5hk1, chain A).^6^ The system was prepared using the Protein Preparation Wizard tool, from the Schrodinger Suite (2024-3),^66,67^ by adding missing hydrogen atoms, reconstructing incomplete side-chain groups, building unresolved loops, assigning favorable protonation states of ionizable amino acids at physiological pH and performing a minimization with the OPLS4 force field.^68^ Compounds 1 (CHEMBL1917704) and 2 (CHEMBL1783464) were prepared to be docked using the LigPrep tool,^69^ which generates all the possible ionization states and tautomers at a pH value of 7.0 ± 2.0 using the OPLS4 force field. The LigPrep output was employed for docking simulations performed by Grid-based ligand docking with energetics (GLIDE).^70–74^ We adopted the standard precision (SP) protocol with all default settings, using a cubic grid having an edge of 10.0 Å for the inner box and 26.5 Å for the outer box, and centered on the co-crystallized ligand position.

## 3. Results and discussion

**Fig. 2:**
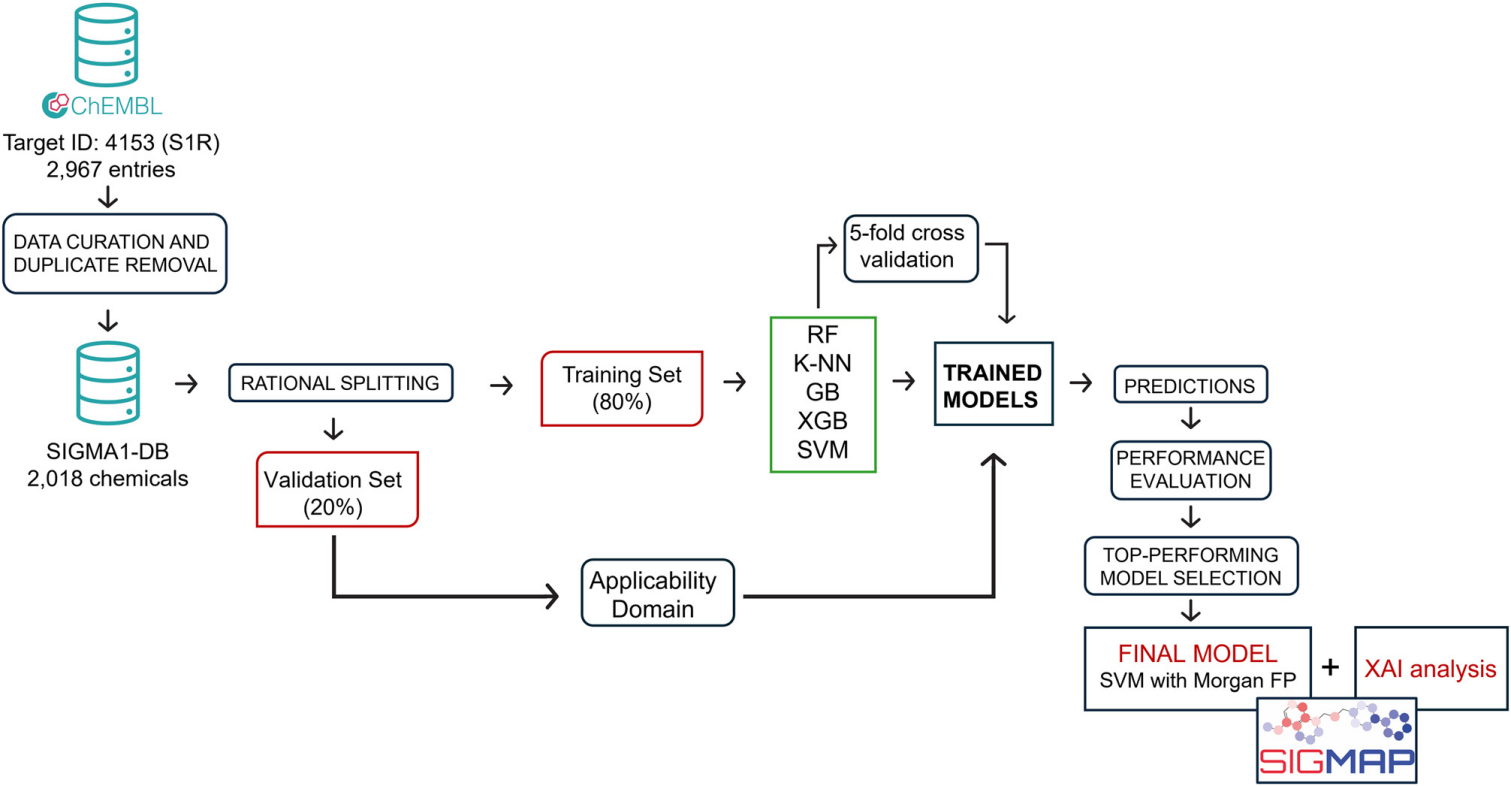
Flowchart showing the main steps of the developed computational workflow.

With the aim of predicting the affinity profile of S1R ligands, 25 classifiers were developed employing a highly-curated dataset (*SIGMA1-DB*) consisting of 2018 compounds with affinity data (*K*_i_) extracted from the ChEMBL v33 database. To this end, five different types of fingerprints (AtomPair, Morgan, MACCS, Torsion and CSFP) were used to characterize each chemical structure within the database, and five machine learning (ML) algorithms, namely RF, KNN, GB, XGB and SVM, were trained and validated using the KNIME Analytics Platform. More specifically, *SIGMA1-DB* was split into a TS and a VS*.* Firstly, we used the TS to perform hyperparameter tuning based on a 5-fold cross-validation (5-CV), and then the models obtained with the best identified parameters were validated using the VS*.* For the sake of clarity, Fig. 2 displays the main steps of the adopted computational workflow. To further assess the predictivity of the best-performing model in practical applications, we employed two external sets (ES1 and ES2). This section will focus on analyzing the key quality metrics (SE, SP, ACC, MCC, AUC and +LR) calculated after the hyperparameter tuning and then for the validation process, aiming to identify the top-performing classifier. Furthermore, we will discuss the results of the two explainability methods based on case studies provided.

### 3.1 5-fold cross validation procedure

Aiming at performing the hyperparameter tuning of the considered ML algorithms and simultaneously challenging their ability to provide predictive classifiers of S1R affinity, we employed the 5-fold cross-validation (5-CV) approach. Table 1 reports the quality metrics computed for each model, trained with the best parameters identified through this process. It is important to point out that the metrics for each model are calculated by averaging the results across five iterations, with different subsets of the TS used for validation in each iteration. Because of this, we would like to emphasize that the accuracy ACC, which is the most comprehensive metric we have presented, shows a standard deviation value below 0.02 across all 25 models, thus strongly supporting their stability. Furthermore, all the models return quality metrics, indicative of good predictive power (Table 1). This is particularly evident when the returned AUC (in most cases ≥0.80) and SE (always ≥0.78) values are considered. Since our objective in this study was to build classifiers for the early stages of a drug discovery (DD) process – rather than for toxicological purposes – we prioritized SP in 5-fold cross validation. Indeed, reducing false positives (*i.e.*, molecules predicted to be able to target S1R with high affinity but later experimentally disproven) is critical to avoid a wasteful investment of time and money in the DD process. For this reason, among the different quality metrics herein considered, we focused our attention on SP and +LR. In particular, while SE and SP exhibit comparable trends across the five model subgroups, SP values are consistently lower than SE values. More specifically, the best performance in terms of SP is achieved when XGB (0.74 when AP is used as the fingerprint) and SVM (0.72 when AP, Morgan and Torsion are employed) are used as algorithms. Furthermore, all classifiers yield interesting +LR scores, particularly when XGB (from 2.25 to 3.19) and SVM (from 2.54 to 2.95) are employed. Based on this early analysis, it appears that the models generated using the XGB and SVM algorithms best align with our goal of minimizing false positives. However, to conduct a more detailed and accurate assessment, it is necessary to implement validation for all the developed models.

**Table 1:** Performances in 5-fold cross-validation (5-CV) of the developed models. For each model, the following statistics are reported: sensitivity (SE), specificity (SP), accuracy (ACC), Matthews correlation coefficient (MCC), area under the ROC (AUC), negative likelihood ratio (−LR) and positive likelihood ratio (+LR)

		SE	SP	ACC	AUC	MCC	−LR	+LR
AtomPair	RF	0.91	0.64	0.79	0.86	0.58	0.14	2.55
	*K*-NN	0.88	0.65	0.78	0.83	0.55	0.18	2.54
	GB	0.87	0.70	0.79	0.87	0.58	0.19	2.87
	XGB	0.84	0.74	0.79	0.85	0.58	0.22	3.19
	SVM	0.83	0.72	0.78	0.76	0.56	0.23	2.95
Morgan	RF	0.87	0.64	0.77	0.83	0.53	0.20	2.41
	*K*-NN	0.86	0.64	0.76	0.82	0.51	0.23	2.38
	GB	0.85	0.70	0.78	0.85	0.56	0.23	2.75
	XGB	0.85	0.70	0.78	0.85	0.56	0.21	2.86
	SVM	0.80	0.72	0.76	0.76	0.52	0.28	2.84
Torsion	RF	0.91	0.60	0.76	0.83	0.53	0.16	2.21
	*K*-NN	0.87	0.63	0.76	0.82	0.51	0.21	2.32
	GB	0.84	0.69	0.77	0.84	0.54	0.23	2.69
	XGB	0.82	0.70	0.77	0.83	0.53	0.25	2.72
	SVM	0.79	0.72	0.76	0.75	0.51	0.30	2.79
MACCS	RF	0.89	0.62	0.77	0.83	0.54	0.18	2.32
	*K*-NN	0.86	0.64	0.76	0.80	0.51	0.23	2.39
	GB	0.82	0.69	0.76	0.83	0.51	0.27	2.64
	XGB	0.85	0.70	0.78	0.84	0.56	0.22	2.84
	SVM	0.78	0.69	0.74	0.74	0.48	0.31	2.55
CSFP	RF	0.85	0.63	0.75	0.80	0.50	0.24	2.31
	*K*-NN	0.84	0.62	0.74	0.79	0.48	0.25	2.22
	GB	0.85	0.66	0.76	0.82	0.52	0.23	2.46
	XGB	0.82	0.64	0.73	0.78	0.46	0.29	2.25
	SVM	0.79	0.69	0.74	0.74	0.48	0.30	2.54

### 3.2 Validation

**Fig. 3:**
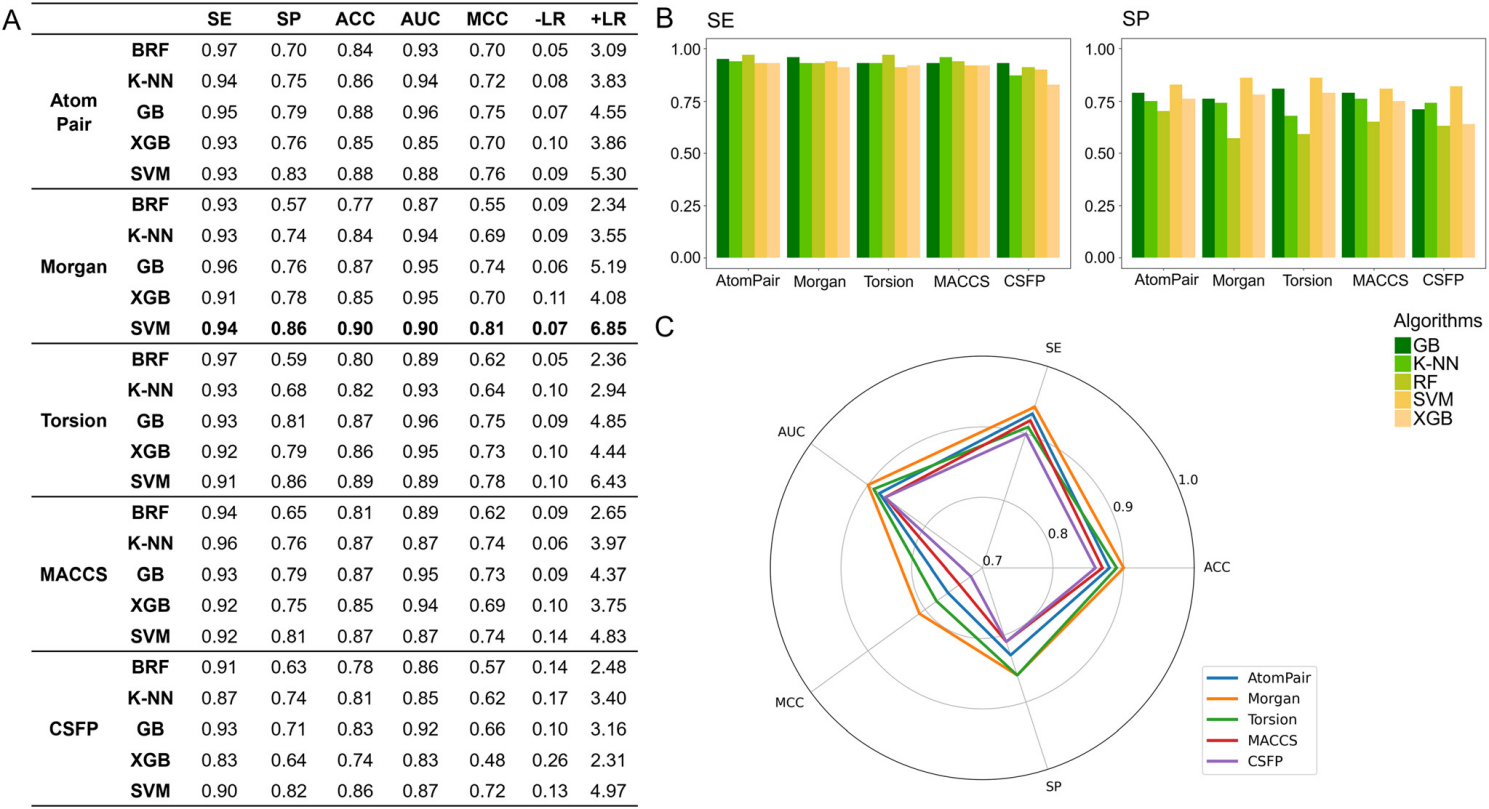
Performances in validation: A) quality metrics computed for all the developed models; B) histograms displaying the computed SP and SV values for all the classifiers; C) radar plot comparing the performance of the models developed using SVM as the algorithm. RF: random forest; *K*-NN: *K*-nearest neighbors; GB: gradient boosting; XGB: extreme gradient boosting; SVM: support vector machine; SE: sensitivity; SP: specificity; ACC: accuracy; AUC: area under the ROC; MCC: Matthews correlation coefficient; −LR: negative likelihood ratio; +LR: positive likelihood ratio.

To identify the best-performing model, all 25 developed classifiers were subjected to internal validation using the VS obtained from the *SIGMA1-DB* split, as detailed in the Materials and methods section, and comprising 403 compounds. As a first step, all compounds underwent the AD filter. Interestingly, none were excluded, confirming that the VS can be reasonably considered representative of the TS due to the rational split performed. As shown in Fig. 3A, this validation confirmed the trend already observed in the 5-CV procedure, with all models achieving high SE and AUC values, with the latter exceeding 0.95 in some cases. It is worth noting, for example, the high AUC values obtained by the GB algorithm (0.96) when using both AP and Torsion fingerprints. Moreover, as returned by the 5-CV approach, significant differences can also be found between SE and SP values, with the former being consistently higher than the latter (Fig. 3B). Specifically, except for models developed using CSFP fingerprints, SE values consistently exceeded 0.90. Less uniformity in the performance of the considered algorithms is instead observed when considering SP values, ranging from 0.57 (model obtained using the Morgan fingerprint and the RF algorithm) to 0.86 (model obtained using the same fingerprint but with SVM as the algorithm). More generally, regardless of the fingerprints considered, SVM consistently yields the highest SP values (between 0.81 and 0.86). Data analysis further indicates that the performance of various fingerprints is roughly similar, except for CSFP which generally performs less well. This holds true whether observing SP or SE values. Remarkably, the SVM-based classifiers return also the highest MCC values (ranging from 0.72 to 0.81), irrespective of the considered fingerprint thus suggesting that this algorithm better performs among the five considered. To select the top-performing model, we compared the performance of all SVM-based classifiers. Fig. 3C reports a radar plot itemizing the computed quality metrics for these models. The SVM-based model developed using the Morgan fingerprint outperformed the others, achieving satisfactory values for sensitivity (SE = 0.94), specificity (SP = 0.86), accuracy (ACC = 0.90), area under the curve (AUC = 0.90), Matthews correlation coefficient (MCC = 0.81), and positive likelihood ratio (+LR = 6.85). For these reasons, it was chosen as the best-performing model.

Finally, to further evaluate the robustness of the top-performing model, we conducted an additional validation using two external datasets (ESs), one consisting of 46 (ES1) and the other consisting of 39 (ES2) compounds. Following the protocol already established for dataset preparation, we extracted all entries classified under the target ID (CHEMBL4153) associated with S1R and annotated exclusively with *K*_i_ measures from the ChEMBL v34 database to conduct a temporal validation of the best-performing model. Using the same semi-automated procedure described in the ‘Dataset preparation’ section, after duplicates and compounds already in the *SIGMA1-DB* were removed, we were left with a set of 52 compounds. As detailed in the ‘Applicability domain’ section, a filter was applied to the set leading to the exclusion of 6 compounds. The process resulted in the ES1 dataset consisting of 46 compounds, comprising 35 S(+) and 11 S(−).

To construct the ES2 dataset, 39 compounds were extracted from two studies by Dichiara *et al.*^75^ and Szczepańska *et al.*^76^ and then underwent the same preprocessing steps as ES1, including the application of the ‘Applicability Domain’ filter. Notably, all 39 compounds (33 S(+) and 6 S(−)) fell within the applicability domain. The model successfully predicted 34 out of 46 compounds for ES1 and 27 out of 39 for ES2, further demonstrating its real-world applicability.

### 3.3 Explainability analysis

**Fig. 4:**
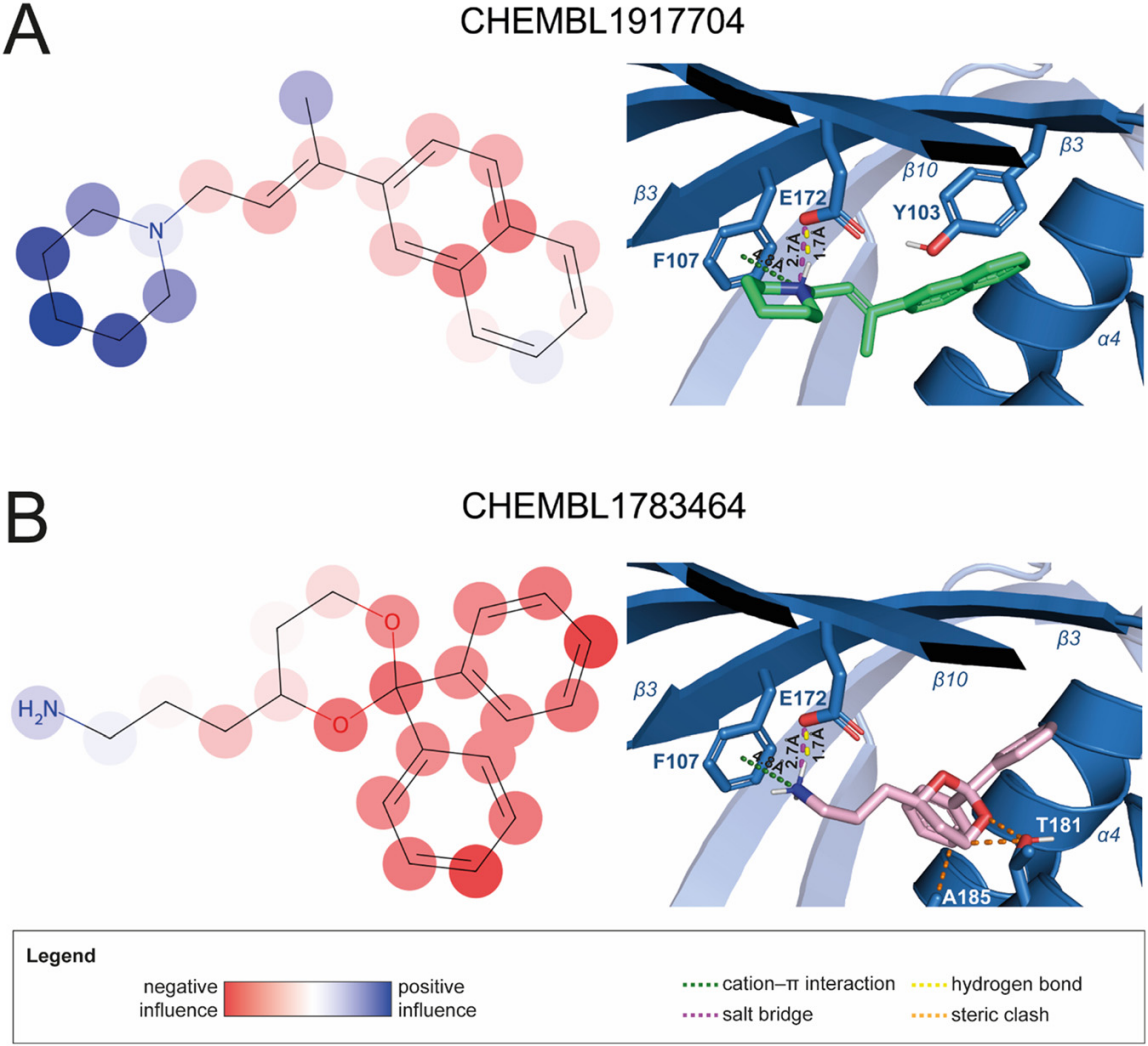
SHAP analysis and docking simulations performed on two compounds belonging to the VS: CHEMBL1917704 (A), experimentally proved to be a high-affinity S1R binder and CHEMBL1783464 (B), experimentally proved to be a low-affinity S1R binder. The left panel depicts the substructures predicted to positively (negatively) influence ligand affinity. The docking poses and the relevant ligand–protein interaction are shown in the right panel.

To enhance the clarity, transparency, and interpretability of the selected model, we implemented a local *post-hoc* XAI method known as SHAP. This analysis provides insights into the reasons behind predictions, specifically how each bit of the fingerprint contributes to the output. Given that the most effective model was trained using molecules described by Morgan fingerprints, we can leverage this analysis to elucidate how each substructure of a molecule (each represented by one or more bits) affects the prediction. Fig. 4 displays the results of the SHAP analysis and of the protein docking for the two molecules from the VS which are correctly predicted by the model. Notice that substructures predicted to positively (negatively) influence ligand affinity are highlighted in blue (red). For instance, it is possible to note that in compound 1 (CHEMBL1917704), which is experimentally known to be a potent S1R binder,^77^ the piperidine moiety is highlighted in blue, likely due to the presence of the nitrogen atom. This observation aligns with existing pharmacophoric models, which consistently identify the presence of positive ionizable groups (*i.e.*, a basic amino group of piperidine) as crucial for ligand–receptor interaction with S1R.^21^ The importance of this structural feature is also evident in the representation of compound 2 (CHEMBL1783464), displaying low affinity towards the S1R receptor.^78^ Despite most substructures being highlighted in red, in line with the negative prediction, the amino group remains blue, indicating that the model accurately recognizes the importance of this group for ligand interaction with S1R.

To further validate the results of the SHAP analysis, we proceeded to dock compounds 1 and 2 into the S1R binding pocket. The difference in affinity between the two ligands is reflected by their computed docking score: compound 1, which is a potent S1R binder, returns a score of −9.140 kcal mol^−1^, while compound 2 returns a score of −7.301 kcal mol^−1^. The importance of the protonated amino group of both molecules, which is highlighted by the SHAP analysis as a key feature promoting binding, is corroborated by the docking results. For both compounds, this group forms a strong salt bridge reinforced by a hydrogen bond with E172. Furthermore, the protonated moiety engages in a cation–π interaction with F107. The atoms of compound 2 predicted to be unfavorable for binding by the SHAP analysis correspond to steric clashes in the docking pose, specifically with residues T181 and A185. The alignment between the SHAP values and docking results strengthens the reliability of our explainability analysis, as both approaches consistently identify the key interactions influencing binding affinity.

By implementing a second explainability analysis known as Contrastive Explanation, we can further test the robustness and stability of the predictions. This analysis involves generating analogues of the input compound as a first step; then these analogs are subjected to prediction by the classifier, producing similar (same prediction) and dissimilar (counterfactual) examples.^61^ For instance, Table 2 illustrates all the analogs generated for compounds 1 and 2 (shown in Fig. 4) along with the corresponding model prediction. The results demonstrate consistent behavior: all the compounds generated from compound 1 (S(+)) are predicted positively by the model, while those generated from compound 2 (S(−)) are predicted negatively, each with high prediction probabilities. These results strengthen the robustness of the classifier, indicating that the model remains stable in its predictions even when small structural modifications are made to the input molecule. Noteworthily, the Contrastive Explanation can also lead to the generation of counterfactuals, making this analysis also a valuable tool for identifying the structural changes necessary to modify the predictions and therefore, obtain valuable clues for design.

Contrastive Explanation-based analysis performed on two compounds belonging to the VS: CHEMBL1917704 (1), experimentally proved to be a high-affinity S1R binder and CHEMBL1783464 (2) experimentally proved to be a low-affinity S1R binder

**Table:** 

Analogues	Prediction	High affinity probability	Analogues	Prediction	High affinity probability
1 S(+)					
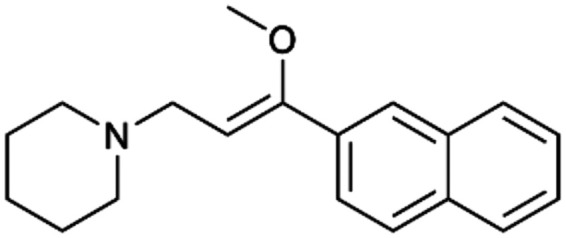	S(+)	0.74	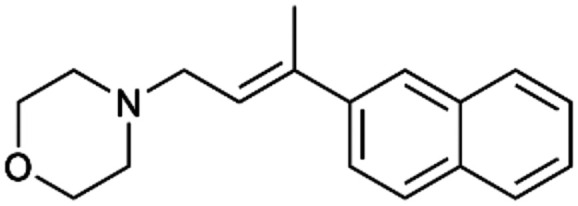	S(+)	0.87
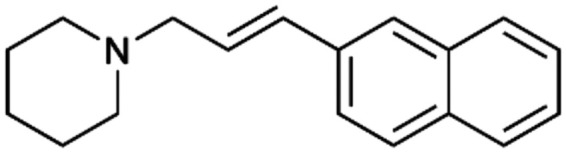	S(+)	0.73	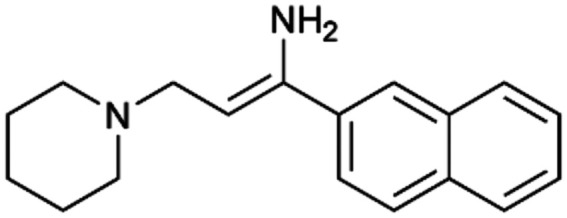	S(+)	0.68
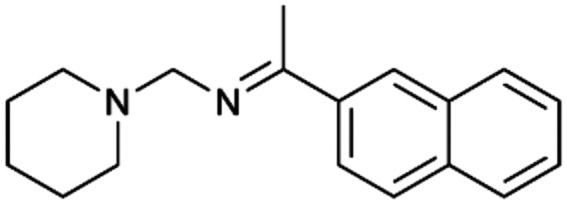	S(+)	0.92	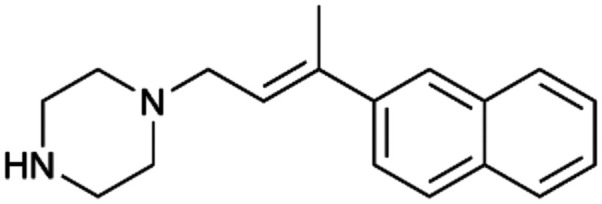	S(+)	0.77
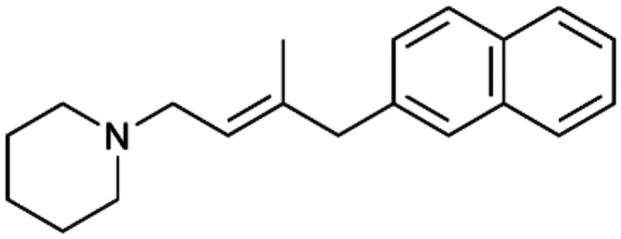	S(+)	0.77	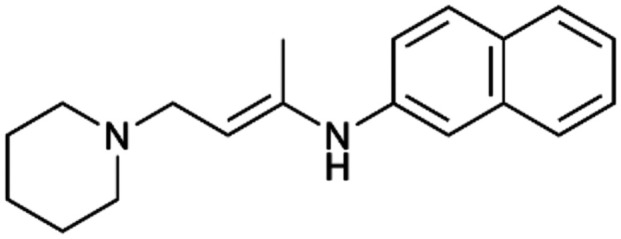	S(+)	0.59
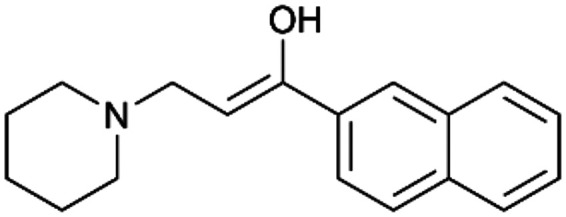	S(+)	0.74	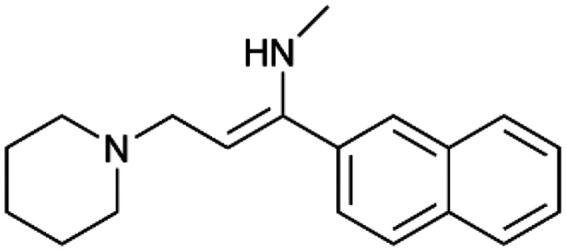	S(+)	0.81

**Table:** 

2 S(−)					
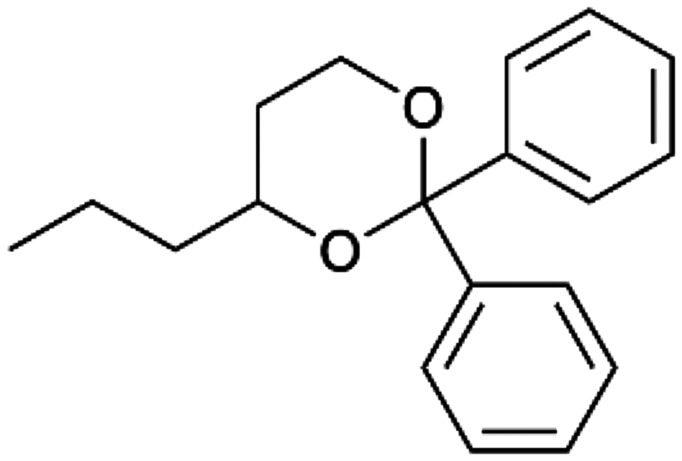	S(−)	0.17	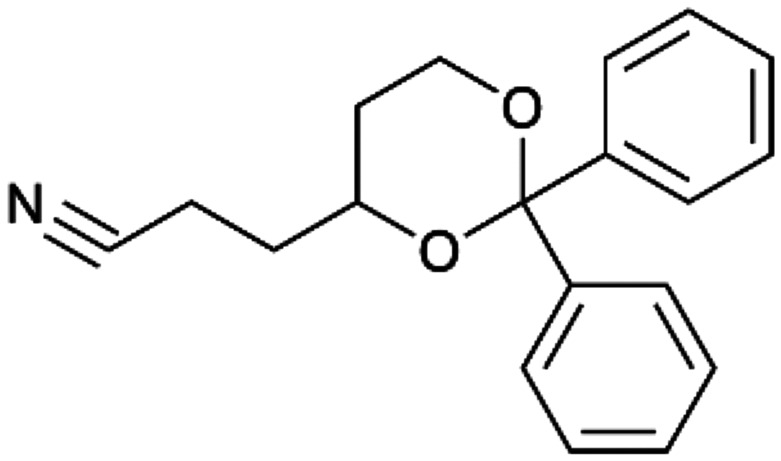	S(−)	0.09
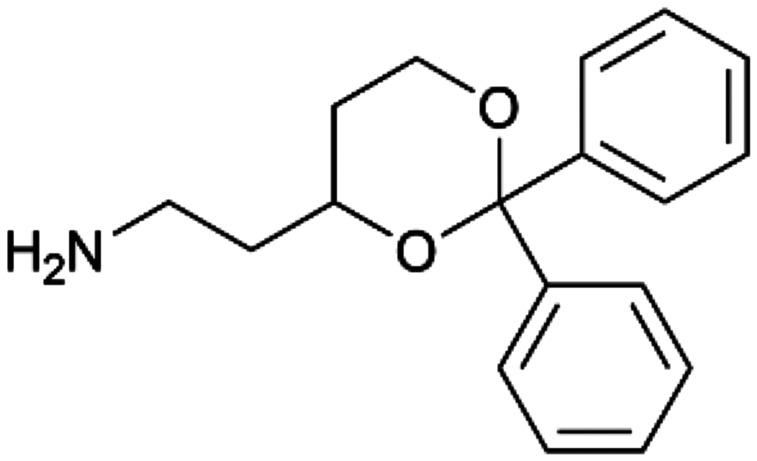	S(−)	0.04	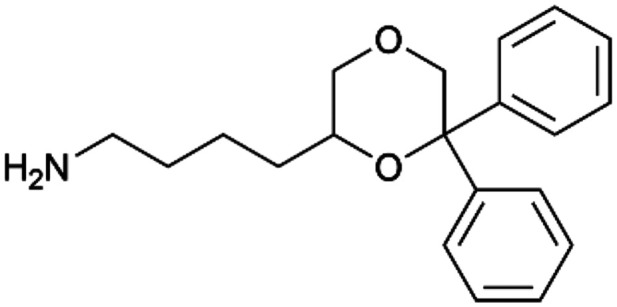	S(−)	0.04
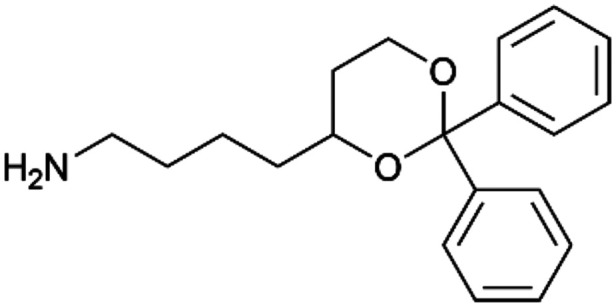	S(−)	0.04	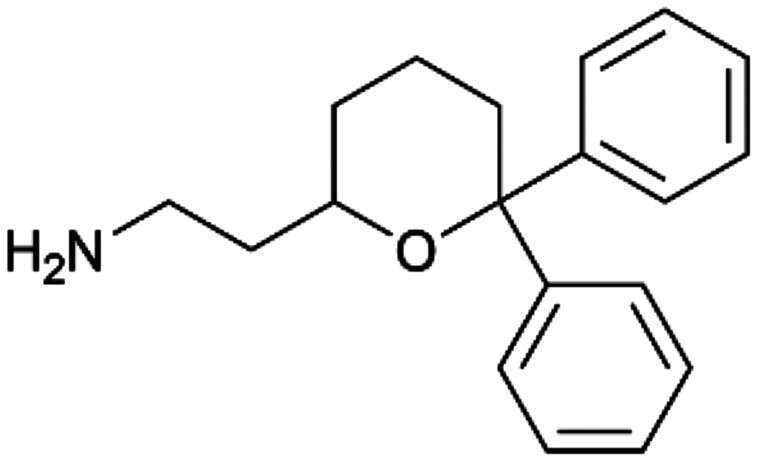	S(−)	0.12
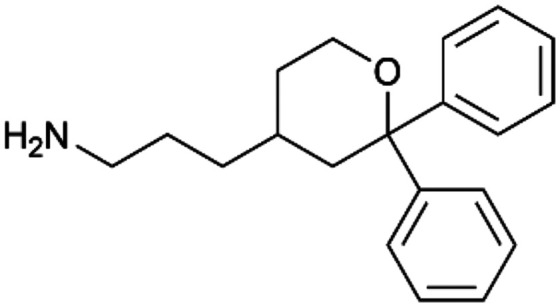	S(−)	0.16	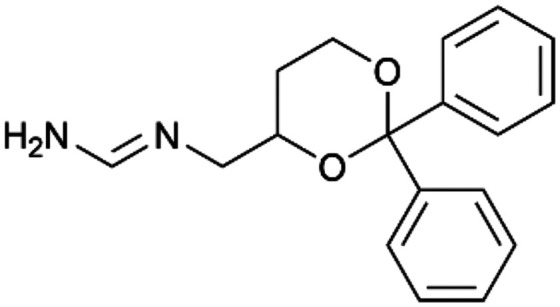	S(−)	0.08
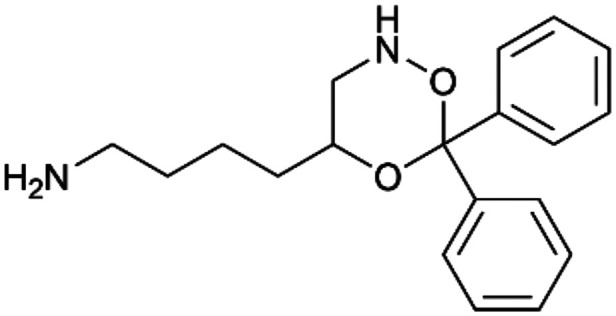	S(−)	0.13	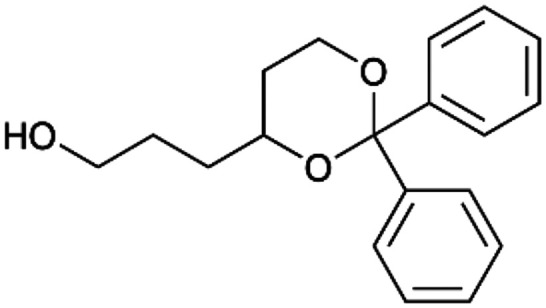	S(−)	0.08

## 4. SIGMAP: a freely accessible web platform

**Fig. 5:**
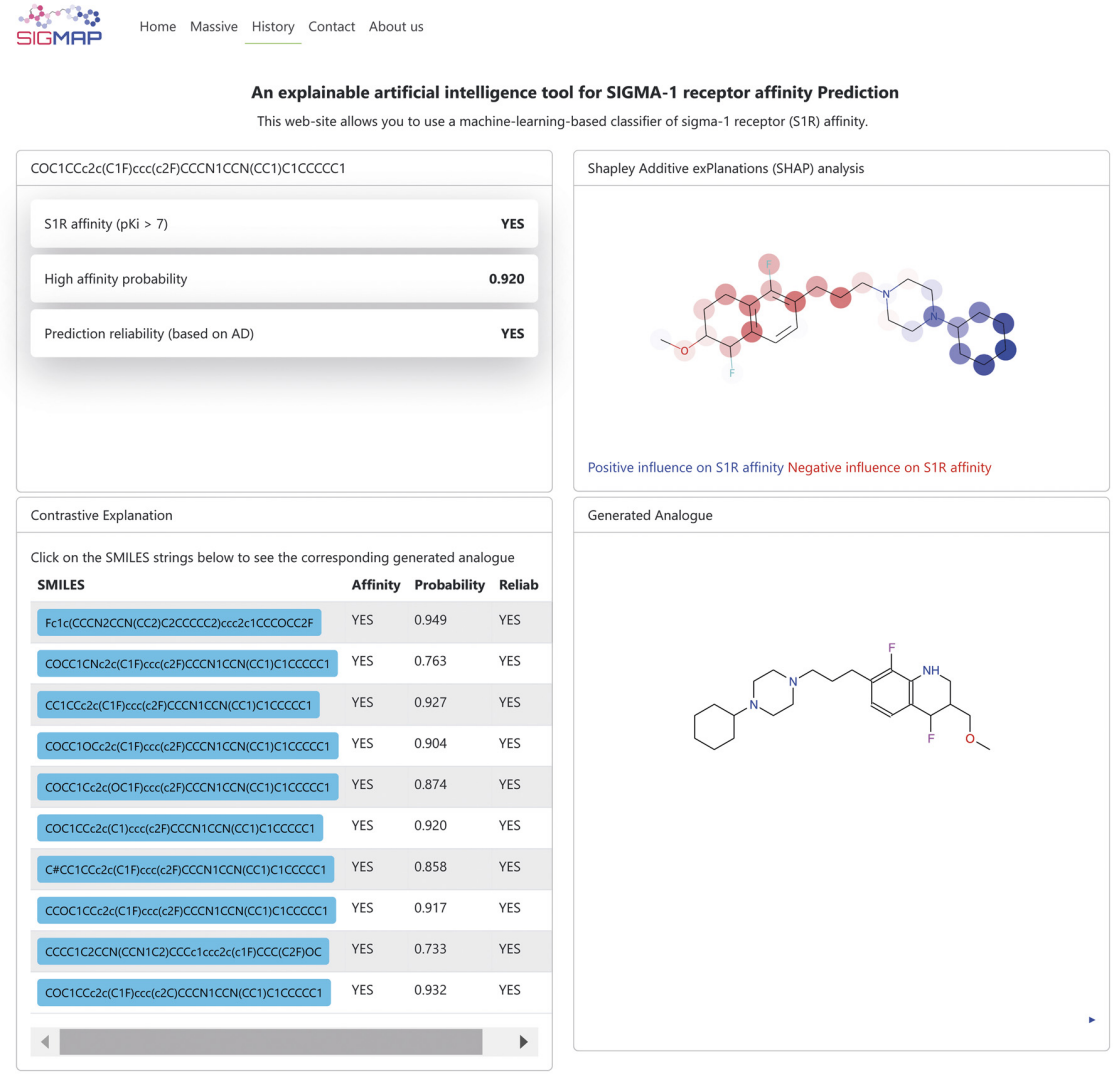
Example of the output page returned by the SIGMAP web platform.

In this work we present SIGMAP, a freely accessible web platform that hosts our top-performing classifier, based on the SVM algorithm and Morgan fingerprints. The platform is designed to facilitate virtual screening and allows users to submit their query molecules in two main ways: either by drawing the 2D structure of their molecule using the integrated JSME canvas applet^79^ or by directly inputting the SMILES string of the molecule into the designed text field. For batch processing, users can upload a text file containing a list of SMILES strings by accessing the “Massive” section. Once the input is submitted, SIGMAP generates predictions regarding the affinity of each compound toward the S1R receptor. The prediction results are displayed as “YES” if the classifier predicts high S1R affinity and “NO” if no affinity is predicted. In cases of high affinity, a prediction confidence, estimated by the KNIME predictor node, is also provided. Additionally, the platform provides information on the reliability of the predictions, based on the AD of the model. When the query molecule is unique, SIGMAP also provides the user with the results of both SHAP and Contrastive Explanation-based analyses. Fig. 5 illustrates an example of such an output. The top left section displays affinity prediction results along with prediction confidence and reliability. The top right section, labeled “Shapley Additive exPlanations (SHAP) analysis”, presents the SHAP analysis output, highlighting the molecule in blue and red to indicate whether specific substructures positively or negatively affect the affinity prediction toward S1R, respectively. Additionally, in the bottom left section, identified with the “Contrastive Explanation” label, the user can explore the SMILES strings of the generated molecules, each accompanied by affinity predictions toward S1R, as well as their prediction confidence and reliability. By clicking on a SMILES button, the corresponding molecule is displayed in the “Generated analogue” section in the bottom right section. Users can also download the generated output as a text file. Noteworthily, a link to download the output is sent directly to the user's registered email address. The web platform also features a “History” page, which keeps a detailed log of all user executions. This page preserves both the input SMILES files and their corresponding output, ensuring that users can easily access past results.

## 5. Conclusions

In recent decades, machine learning approaches have revolutionized medicinal chemistry, offering fast and reliable tools that significantly enhance and support the development of biologically active compounds. Despite the crucial importance of S1R in various areas of pharmacological research, there was a lack of explainable intelligent systems to assist chemists in designing new ligands with potential affinity for this receptor. To bridge this gap, we developed several machine learning models able to predict S1R ligand affinity. These models utilized five classification algorithms (RF, *K*-NN, GB, XGB, and SVM) and five different molecular fingerprints (AtomPair, Morgan, MACCS, Torsion, and CSFP) for characterizing a dataset (*SIGMA1-DB*) comprising 2018 rigorously curated compounds extracted from ChEMBL v33. Through a comprehensive validation procedure, the SVM algorithm emerged as the top performer, achieving ACC and AUC values of 0.90 when using Morgan as the fingerprint. To enhance the interpretability of the classifier's predictions, we implemented two independent XAI approaches: SHAP and Contrastive Explanation. Notably, by employing Contrastive Explanation analysis, we can generate analogs predicted to outperform the starting query, thereby providing valuable insights for designing new and more potent S1R ligands. The top-performing model and the XAI analyses are available through a user-friendly web platform called SIGMAP, developed by our research team. To the best of our knowledge, SIGMAP (https://www.ba.ic.cnr.it/softwareic/sigmap/) is the first freely accessible tool that integrates high predictive accuracy with transparent and interpretable insights, specifically designed to predict the S1R affinity of drug candidates.

## Data availability

The following data are made available in the ESI:†

- Table reporting the parameters optimized for each trained model based on the hyperparameter tuning performed on a 5-CV.

- TS_SIGMA1-DB excel file containing the 1615 SMILES strings (and the corresponding experimental values) of the chemicals belonging to the SIGMA-DB dataset and used as a training set.

- VS_SIGMA1-DB excel file containing the 403 SMILES strings (and the corresponding experimental values) of the chemicals belonging to the SIGMA-DB dataset and used as validation.

- ES1 excel file containing the 46 SMILES strings (and the corresponding experimental values) of the chemicals used as an external set.

- ES2 excel file containing the 39 SMILES strings (and the corresponding experimental values) of the chemicals used as an external set.

SIGMAP is freely available in a GitHub repository (https://github.com/alberdom88/SIGMAP).

## Author contributions

MCL: investigation, methodology, software, writing – original draft; NC: software; VN: methodology, writing – review & editing; AP: writing – review & editing; software; MS: writing – review & editing, funding acquisition; RL: writing – review & editing; software; CA: writing – original draft, conceptualization; DA: investigation, methodology, software, writing – original draft, supervision; GFM: conceptualization, funding acquisition, methodology, supervision, writing – original draft.

## Conflicts of interest

There are no conflicts of interest to declare.

## Supplementary Material

MD-016-D4MD00722K-s001

MD-016-D4MD00722K-s002

MD-016-D4MD00722K-s003

MD-016-D4MD00722K-s004

MD-016-D4MD00722K-s005
