# Supplementary material for: SIGMAP: an explainable artificial intelligence tool for SIGMA-1 receptor affinity prediction
Source: RSC Med Chem. 2024 Nov 8;16(2):835–48. doi: 10.1039/d4md00722k (PMC11605305; doi:10.1039/d4md00722k)
Supplement: MD-016-D4MD00722K-s003 [file MD-016-D4MD00722K-s003.pdf]

Supplementary Information (SI) for RSC Medicinal Chemistry.
This journal is © The Royal Society of Chemistry 2024

SIGMAP: an explainable artificial intelligence tool for SIGMA-1 receptor affinity Prediction

Maria Cristina Lomuscio[a], Nicola Corriero[b], Vittoria Nanna[b], Antonio Piccinno[c], Michele Saviano[d], Rosa Lanzilotti[c], Carmen Abate[b,e], Domenico Alberga[b] and Giuseppe Felice Mangiatordi[b*]*

[a] Dipartimento di Medicina di Precisione e Rigenerativa e Area Jonica (DiMePRe-J), Università degli Studi di Bari Aldo Moro, Piazza Giulio Cesare, 11, Policlinico, 70124, Bari, Italy
[b] CNR – Institute of Crystallography, Via Amendola 122/o, 70126 Bari, Italy
[c] Department of Computer Science, University of Bari "Aldo Moro", via E. Orabona, 4, I-70125 Bari, Italy
[d] CNR – Institute of Crystallography, Via Vivaldi 43, 81100, Caserta, Italy
[e] Department of Pharmacy – Pharmaceutical Sciences, University of Bari "Aldo Moro", via E. Orabona, 4, I-70125 Bari, Italy

SUPPLEMENTARY MATERIAL

Table S1. Parameters optimized for each trained model based on the hyperparameter tuning performed on a 5-CV.

Table S1

Algorithm	Optimized parameters	AtomPair	Morgan	Torsion	MACCS	CSFP
**RF**	split criterion	gini index	gini index	gini index	gini index	gini index
	attribute sampling	square root	square root	square root	square root	square root
	set of attributes for each tree	different	different	different	different	different
	number of trees	205	123	235	217	162
	tree depth	10	9	9	9	10
	equal size sampling	yes	yes	yes	yes	yes
**KNN**	number of neighbors to consider	3	5	3	5	5
	weight neighbors by distance	yes	yes	no	yes	yes
**GB**	number of trees	463	481	424	378	29
	learning rate	0.017	0.014	0.017	0.004	0.074
	attribute sampling	square root	square root	square root	square root	square root
	set of attributes for each tree	different	different	different	different	different
	maximum tree depth	8	9	17	23	17
**XGB**	eta	0.289	0.302	0.306	0.151	0.674
	boosting rounds	162	339	203	180	298
	gamma	0.621	0.481	0.334	0.315	2.405
	lambda	3.912	4.019	0.604	4.556	7.31
	alpha	0.411	0.409	9.061	0.411	1.258
	maximum depth	3	6	6	6	7
**SVM**	kernel	RBF	RBF	RBF	RBF	RBF
	nu	0.1	0.1	0.1	0.3	0.3
	gamma	0.02	0.02	0.02	0.32	0.07
